# Thermomechanical and mechanical analysis of polylactic acid/polyhydroxyalkanoate/poly(butylene succinate-*co*-adipate) binary and ternary blends†

**DOI:** 10.1039/d4ra05684a

**Published:** 2025-01-06

**Authors:** Alisa Sabalina, Oskars Platnieks, Gerda Gaidukova, Arturs Aunins, Toms Valdemars Eiduks, Sergejs Gaidukovs

**Affiliations:** a Institute of Chemistry and Chemical Technology, Faculty of Natural Sciences and Technology, Riga Technical University P. Valdena 3 LV-1048 Riga Latvia oskars.platnieks_1@rtu.lv sergejs.gaidukovs@rtu.lv; b Institute of Materials and Surface Engineering, Faculty of Natural Sciences and Technology, Riga Technical University P. Valdena 3 LV-1048 Riga Latvia

## Abstract

Research efforts are increasingly directed towards the development of biodegradable polymers derived from renewable agricultural resources. Polymer blends, which combine multiple polymers, offer enhanced properties such as ductility and toughness while being more cost-effective compared to the development of specialized copolymers. This study examines nine binary and four ternary blends of polylactic acid (PLA), poly(butylene succinate-*co*-adipate) (PBSA), and polyhydroxyalkanoate (PHA). The morphology of most blends was characterized by spherical inclusions. Binary blends with a 50/50 wt% ratio demonstrated distinctly different interactions between the bioplastics. The addition of PBSA increased both tensile elongation and impact strength. Notably, the PLA/PBSA blend with a 30/70 wt% ratio exhibited an elongation at break of 172%. In blends where PBSA constituted 30 wt%, there was an increase in impact strength, from 15% to 29% in PHA/PLA and PLA/PBSA blends, respectively. Overall, PBSA exhibited better compatibility with PLA compared to PHA. Mechanical testing of ternary blends was integrated with the binary blends' results to construct ternary diagrams. Dynamic mechanical analysis revealed that the glass transition temperatures in PBSA binary blends remained largely independent of the blend composition. Calorimetric analysis revealed a shift in crystallization behavior. This research highlights the potential of biopolyester blends in developing bioplastics with tailored mechanical properties for applications in automotive, agriculture, food packaging, and shape memory materials.

## Abstract 2

Research is increasingly focused on developing biodegradable polymers from renewable agricultural resources. Polymer blends, combining multiple polymers, deliver improved ductility and toughness while remaining cost-effective compared to specialized copolymers.

## Graphical abstract

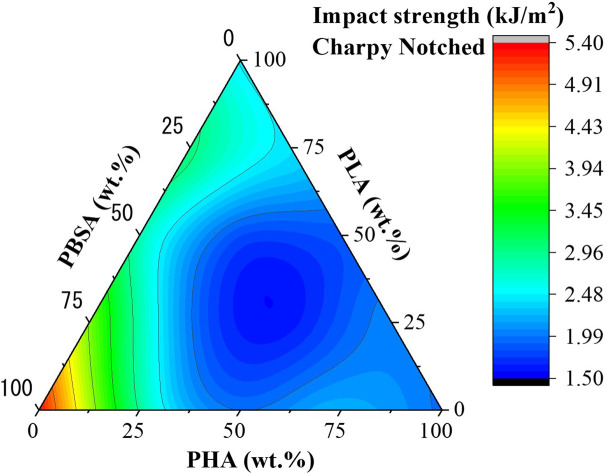

## 1. Introduction

Petroleum-based polymers have become an essential part of the modern world. However, the limited availability of oil resources and fluctuating oil prices are causing concern for the long-term production of synthetic polymers. Additionally, the environmental impact of petroleum-based polymers as non-degradable waste is a significant concern.^1^ To address these issues, research focuses on developing biodegradable polymers from renewable agricultural resources.^2^ Fossil-based polymers offer a large selection of grades with a wide range of properties, but the options for biodegradable polymers are limited to a small number per producer.^3^

Polymer blends are combinations of two or more polymers, which can enhance properties such as strength, toughness, and thermal stability.^4^ In many cases, the cost of the final product is an essential factor to consider, particularly in commodity and engineering applications, in addition to the quality of the final product. Polymer blends offer a range of options and is cost-efficient, whereas copolymer synthesis is geared towards specialized applications where cost is less of a concern or in cases where production volumes can offset development costs.^5,6^

Biodegradable and/or bio-based industrial polymers, known as bioplastics, have recently gained attention as a potential replacement for traditional commodity plastics. These polymers, such as polylactic acid (PLA), poly(butylene succinate) (PBS), and polyhydroxyalkanoate (PHA), aim to be more environmentally friendly. PLA, which has a relatively high market share of around 20%, is known for its high elastic modulus and tensile strength but can be brittle and lack toughness.^7,8^ To improve these properties, it is often blended with other polymers or plasticizers.^9^ PHA is produced through a unique microbial synthesis mechanism, which grants excellent biodegradability and tunable mechanical properties.^10^ However, its commercial form can have poor melt processability and relatively high price.^11^ PBS and its copolymers, such as poly(butylene succinate-*co*-adipate), are alternatives to softer commodity plastics like polyethylene and polypropylene, with relatively high elongation values and good impact resistance.^12^ PBSA has a relatively low melting temperature of around 80 °C, which can be considered limiting for some applications, while an advantage in terms of energy consumption for processing.^13^

Chen *et al.* reported PLA/polycaprolactone (PCL) blends with various polymer ratios.^14^ Adding PCL enhanced elongation values but reduced elastic modulus and tensile strength values; a notable exception was a ratio of PLA/PCL 80/20, which showed an increase in all tensile properties compared to PLA. Arrieta *et al.* reported PLA/poly(hydroxybutyrate) (PHB) blends and the use of various interfacial compatibilization methods.^15^ The authors reported a significant decrease in mechanical properties for PLA/PHB blends compared to neat polymers. If interfacial compatibilization was applied, then properties could be significantly enhanced. Su *et al.* briefly reviewed the PLA/PBS blends.^16^ The authors noted that the immiscible nature of polymers yields significantly different properties based on the preparation method and that PBS can be used to enhance PLA toughness. Mittal *et al.* reported a rather interesting ternary blend system of PLA/PC/thermoplastic starch (TPS).^17^ Adding PCL enhanced elongation values at the cost of modulus and tensile strength, while TPS did not yield mechanical benefits but could provide increased biodegradability. PLA/poly(butylene-adipate-*co*-terephthalate) (PBAT)/polyolefin elastomer grafted with glycidyl methacrylate was reported as an efficient route to increase elongation and toughness for blends with PLA content around 90 wt%.^18^ While the method was efficient, the composition was only about 90% biodegradable. Nofar *et al.* examined PLA/PBSA/poly(butylene adipate-*co*-terephthalate) (PBAT) blends.^19^ The authors reported that in ternary systems, the hindrance effect of PBSA on the crystallization of PBAT combined with relatively weak interactions with PLA limited the mechanical performance of blends. In addition, the authors noted that using a binary blend of PLA/PBAT was more efficient for enhanced ductility.^20^

The last two decades have seen increasing interest in bio-based and biodegradable polymer blends. As the most competitive solution to conventional polymers, PLA has seen the most reports in this field. Ternary systems are still relatively underexplored and can yield many unforeseen interactions. For this research, two glassy polymers with relatively similar properties, PLA and PHA, were chosen, and a ductile soft PBSA was used to enhance and modify the ductility and toughness. Tensile, impact and dynamical mechanical testing was performed to characterize the mechanical performance of produced blends. The density measurements were used as a control for structural defects and voids. Three selected bioplastics in the research can be applied for agricultural uses, food packaging, and biomedical solutions.

## 2. Materials and methods

### 2.1. Materials

Poly(butylene succinate-*co*-adipate) (PBSA) FD92PM pellets were acquired from PTT MCC Biochem Co., Ltd (Bangkok, Thailand). PBSA is categorized as a semi-crystalline thermoplastic polyester, possessing a 1.24 g cm^−3^ density and a melt flow index (MFI) of 4 g/10 min (measured at 2.16 kg and 190 °C). This material is entirely biodegradable and demonstrates partial bio-based composition, comprising 20–50% bio-derived content, as confirmed by DIN certification 8C083. The polyhydroxyalkanoate (PHA) grade PHI002 was acquired from Nature Plast. PHA is more than 90% biobased (according to ASTM D6866 standard) and is industrially compostable following ASTM D6400 standards. Its melting temperature ranges from 170 to 176 °C, with a melt flow index of 5–10 g/10 min (measured at 2.16 kg and 190 °C) and a 1.23 g cm^−3^ density. Polylactic acid (PLA), marketed under the trademark Ingeo™ and grade 6201D, is manufactured by NatureWorks LLC. It is a 100% bio-based and compostable resin designed explicitly for fiber production. PLA is characterized by a melting temperature of approximately 170 °C, a melt flow index of 15–30 g/10 min (measured at 210 °C, according to ASTM D1238), and a 1.24 g cm^−3^ density. Acetone (HPLC grade, purity ≥99.9%) was sourced from Merck KGaA, Darmstadt, Germany.

### 2.2. Blend preparation

**Fig. 1:**
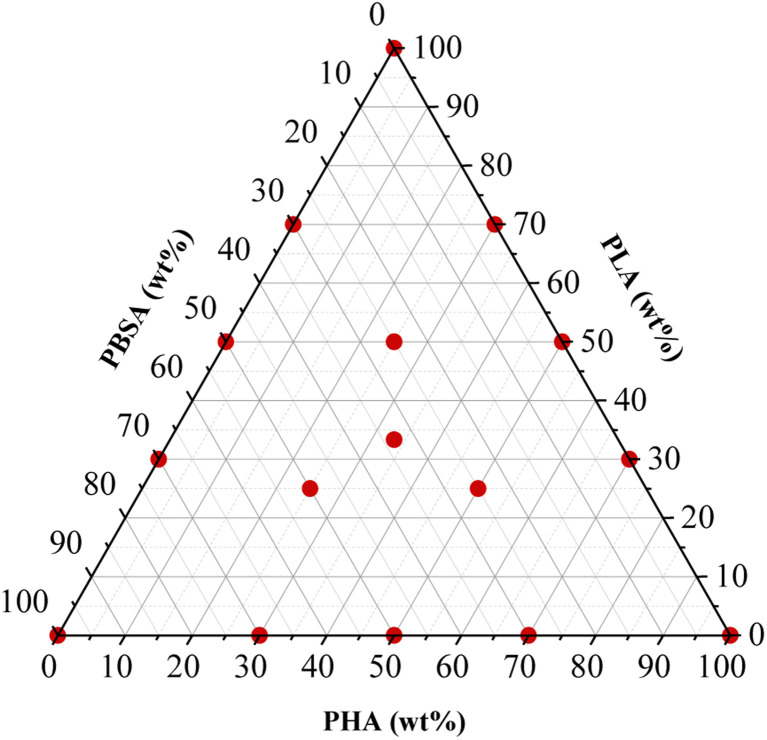
Design of the experiment: sample compositions.

The polymer granules underwent a drying process within a vacuum furnace (J. P. Selecta) at a temperature of 40 °C under a pressure range of 5–20 mbar for 24 hours, preceding subsequent processing steps. Polymer blends were prepared using the Thermo Electron PRISM TSE 16 TC bench-top twin-screw extruder. The compositions of the blends used are detailed in Table 1, while design of the experiment is shown in Fig. 1. During the extrusion process, the barrel temperatures were set as follows: 160 °C in the feeding zone, 165 °C, 170 °C, 175 °C, and 180 °C at the die. The screws were operated at a rotation speed of 24 rpm, and manual feeding of the materials was performed. The extruded polymer strands, with an approximate diameter of 3 mm, were cooled in a water bath and then pelletized to a length of 2 mm using the Thermo Electron PRISM VARICUT 1 equipment. After pelletization, the resulting pellets were subjected to a further drying step in the vacuum furnace at 40 °C, under a pressure range of 5–20 mbar, for 24 h. Finally, the dry pellets were stored in sealed plastic bags.

**Table 1:** Sample abbreviations and compositions

Sample	PHA-A (wt%)	PLA-L (wt%)	PBSA-S (wt%)
PHA	100.0	0.0	0.0
PLA	0.0	100.0	0.0
PBSA	0.0	0.0	100.0
A3L7	30.0	70.0	0.0
A5L5	50.0	50.0	0.0
A7L3	70.0	30.0	0.0
A3S7	30.0	0.0	70.0
A5S5	50.0	0.0	50.0
A7S3	70.0	0.0	30.0
L3S7	0.0	30.0	70.0
L5S5	0.0	50.0	50.0
L7S3	0.0	70.0	30.0
ALS	33.3	33.3	33.3
ALS2	25.0	25.0	50.0
AL2S	25.0	50.0	25.0
A2LS	50.0	25.0	25.0

### 2.3. Sample preparation

The samples comprising polymer blends and pure polymers were fabricated using a BOY 25E injection molding machine. The thermal profile applied for the mixtures in the extrusion area was as follows: 160 °C in the feeding zone, followed by increments to 165 °C, 170 °C, 175 °C, and 180 °C at the die. It is important to note that the thermal profile differed for the PBS and PHA/PBS mixtures. Expressly, the thermal profile for PBS was set at 105 °C, 110 °C, 115 °C, 120 °C, and 125 °C at the die. The thermal profile for PHA/PBS mixtures comprised temperatures of 153 °C in the feeding zone, followed by 158 °C, 163 °C, 168 °C, and 173 °C. The blends were pressed into a mold at a temperature of 40 °C for the blends, PHA, and PLA, while a mold temperature of 20 °C was used for the PBS. During this process, a filling shear rate of 80 1 s^−1^ was applied, with a pressure of 90 bar. The formed samples were then cooled within the mold for a duration of 50 seconds.

### 2.4. Characterization methods

Injection-molded sample cross-cut surface images were obtained using scanning electron microscopy (SEM). Cross-sections were obtained from the fracture surface produced by breaking the samples that had been cooled with liquid nitrogen. The etched surfaces were prepared by immersing the samples in acetone for 24 hours at 20 °C, followed by an additional hour at 30 °C. The FEI Nova NanoSEM 650 Schottky field emission scanning electron microscope (FESEM) was utilized, operating at an acceleration voltage of 10 kV. The samples were mounted on electrically conductive double-sided carbon tape for imaging.

To measure the density (*ρ*), the weight in air and ethanol was determined using Sartorius KB BA 100 electronic scales with a Sartorius YDK 01 hydrostatic density measuring kit. The following equation was used to determine the composite densities:1
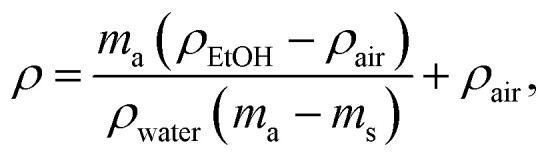
where *m*_a_ is the sample's measured mass in the air; *m*_s_ is the sample's measured mass when submerged in ethanol; *ρ*_EtOH_ is the density of ethanol (0.805 g cm^−3^), measured with the aerometer; *ρ*_air_ is the density of the air (*ρ* = 0.00120 g cm^−3^) and *ρ*_water_ is the density of the water (0.99983 g cm^−3^). For measurements, ten replicates of each sample were utilized.

The tensile properties were determined using a universal testing machine, Tinius Olsen 25ST, according to the ISO 527-2 type 1B standard, with a crosshead speed of 1 mm min^−1^ and an S-type load cell with a capacity of 5 kN. The V-notched Charpy impact test was performed on a Zwick IPM-8 testing machine according to the ISO 179-1 standard.

Dynamic mechanical analysis was performed utilizing a dynamic mechanical analyzer DMA/SDTA861e from Mettler Toledo (USA). The experiment was set up in a dual cantilever deformation configuration and measured a temperature range from −70 °C to 100 °C. The testing protocol entailed a heating rate of 3 °C min^−1^, an applied force of 10 N, a displacement amplitude of 20 μm, and a frequency of 1 Hz. For tests, injection-molded rods were used.

Differential Scanning Calorimetry (DSC) measurements were conducted using a Mettler Toledo DSC-1 analyzer under a nitrogen atmosphere. The thermal analysis involved a heating–cooling–heating cycle over a temperature range of −20 to 220 °C, adding 5 min temperature stabilization after each cycle. The heating and cooling rates were varied at 2.5, 5, 10, 20, and 40 °C min^−1^. Each sample had an average mass of approximately 10 mg. The crystallization behavior of the ternary systems was quantified using eqn (2), as outlined below:2
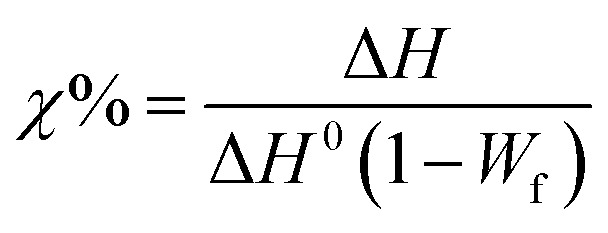
where *W*_f_ is the weight fraction of the second and third components (PHA, PLA, and PBSA) in the blend, Δ*H* is the experimental crystallization enthalpy (J g^−1^) of the polymer in the blends, and Δ*H*^0^ is the melting enthalpy for 100% crystalline polymer taken from the literature. The values of Δ*H*^0^_PHA_ is 146 J g^−1^, Δ*H*^0^_PLA_ is 93 J g^−1^, and PBSA is 110 J g^−1^.^21–23^

## 3. Results and discussion

### 3.1. Blend morphology and compatibility

**Fig. 2:**
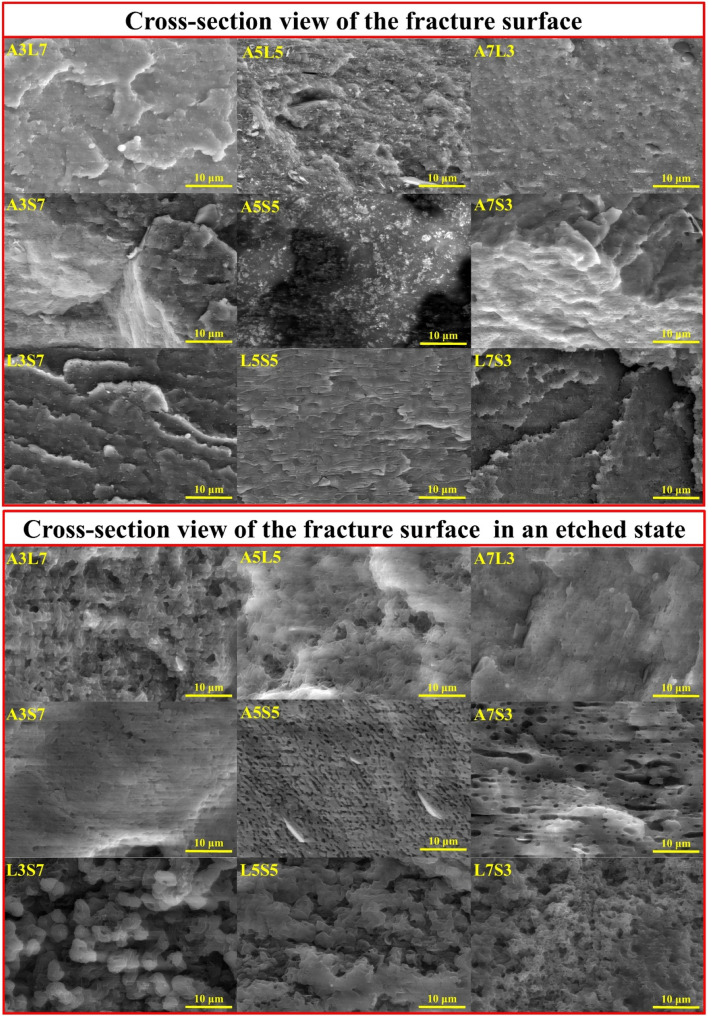
SEM micrographs depicting the cross-sectional views of fracture surfaces and etched surfaces for binary polymer blends.

The SEM micrographs of the cryo-fractured and etched binary blend samples are presented in Fig. 2, while lower magnifications are provided in Fig. S1.† The processing conditions, polymer compatibility, and blend ratio significantly influence the resulting blend morphology, leading to the formation of various structures such as lamellar, droplet-matrix, cylindrical, and co-continuous arrangements.^19^ Microstructural analysis reveals that for most compositions with a 3 : 7 (or 7 : 3) ratio, the minority polymer within the blend adopts a droplet-matrix morphology. A notable exception is the A7S3 composition, which exhibits a hybrid morphology combining lamellar and droplet-type inclusions. The A3L7 blend is characterized by irregular droplet sizes, whereas the L3S7 composition exhibits the largest observed droplet sizes.

In blends with an equal polymer ratio (5 : 5), pronounced morphological differences are evident among the binary compositions. The A5S5 and L5S5 blends display a visibly co-continuous morphology. However, a distinct variation is noted in the size of the morphological features; A5S5 shows smaller structural elements, with some lamellar flakes visible on the etched surface. The A5L5 blend presents a mixed morphology, which is likely due to the proximity of the polymers' melting temperatures. This blend exhibits characteristics of droplet, lamellar, and co-continuous morphologies.

**Fig. 3:**
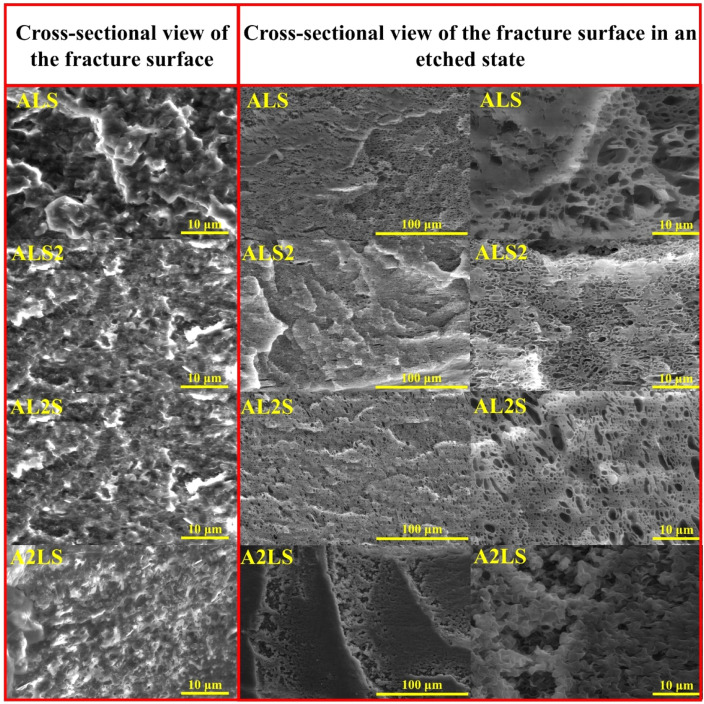
SEM images of the cross-section view of the fracture surfaces in referenced and etched state for ternary ALS, ALS2, AL2S, A2LS blends.

The SEM micrographs of the cryo-fractured and etched ternary blend samples are presented in Fig. 3. Compared to the binary blend samples, the surface of the ternary blend samples appears significantly rougher. Additionally, the pull-out voids in the ternary blends exhibit greater size variability and a more irregular distribution. The etched surfaces offer valuable insights into the morphological characteristics of the blends. The A2LS composition reveals a resemblance of co-continuous and droplet morphologies, while the AL2S blend exhibits a combination of droplet and lamellar structures. In contrast, the ALS2 blend shows a higher prevalence of lamellar-like inclusions. Finally, the ALS composition demonstrates an inconsistent morphology, likely due to the inability of any polymer within the blend to dominate as the majority phase.

The obtained experimental density values for PLA, PHA, PBSA, and blends are presented in Table 2. The three selected biopolymers showed relatively similar values; PHA had the highest density of 1.244 g cm^−3^, and PBSA demonstrated the lowest density of 1.230 g cm^−3^. Thus, measured density values for blends show a relatively small deviation from neat polymers. The binary blends were within the expected combination of density averages with deviations within the margin of error. All ternary blends exhibited decreased density values, indicating potential structural defects, which may arise from compatibility issues or shifts in the crystallinity of the individual components (discussed in the calorimetry section). As PBSA combinations with PHA and PLA show relatively similar trends for mechanical properties (discussed in the next section), it should be noted that blends with PLA have slightly lower densities.

**Table 2:** Density and tensile properties

Sample	*ρ* (g cm^−3^)	*E* (MPa)	*σ*_U_ (MPa)	*ε*_Y_ (%)	*ε*_B_ (%)
PHA	1.244 ± 0.009	2666 ± 254	33.0 ± 2.6	1.5 ± 0.1	1.5 ± 0.1
PLA	1.238 ± 0.009	2545 ± 107	52.7 ± 2.6	2.8 ± 0.2	4.8 ± 1.4
PBSA	1.230 ± 0.003	250 ± 14	19.1 ± 1.3	24.4 ± 2.6	320 ± 25.8
A3L7	1.229 ± 0.007	2218 ± 133	47.2 ± 2.9	2.7 ± 0.2	3.5 ± 0.6
A5L5	1.246 ± 0.005	2620 ± 202	39.3 ± 4.1	1.9 ± 0.1	1.9 ± 0.1
A7L3	1.241 ± 0.003	2687 ± 179	24.9 ± 4.1	1.0 ± 0.1	1.0 ± 0.1
A3S7	1.223 ± 0.009	475 ± 41	16.5 ± 1.5	12.1 ± 1.5	12.1 ± 1.5
A5S5	1.248 ± 0.004	527 ± 61	19.3 ± 1.3	8.2 ± 2.0	10.4 ± 2.9
A7S3	1.259 ± 0.009	1390 ± 60	25.1 ± 0.3	4.3 ± 0.3	4.3 ± 0.3
L3S7	1.232 ± 0.005	418 ± 37	20.3 ± 0.7	19.1 ± 1.1	172 ± 51.2
L5S5	1.233 ± 0.002	1033 ± 110	30.4 ± 3.2	5.8 ± 0.9	8.7 ± 6.0
L7S3	1.235 ± 0.003	1382 ± 86	39.1 ± 1.4	4.0 ± 0.4	15.3 ± 4.3
ALS	1.221 ± 0.006	1598 ± 195	32.6 ± 1.0	3.3 ± 0.1	3.3 ± 0.1
ALS2	1.229 ± 0.005	901 ± 39	24.6 ± 0.4	6.2 ± 0.4	6.2 ± 0.4
AL2S	1.222 ± 0.005	1473 ± 62	35.3 ± 1.6	3.6 ± 0.1	4.4 ± 0.6
A2LS	1.223 ± 0.009	1792 ± 100	27.8 ± 1.6	2.0 ± 0.1	2.0 ± 0.1

### 3.2. Tensile properties

**Fig. 4:**
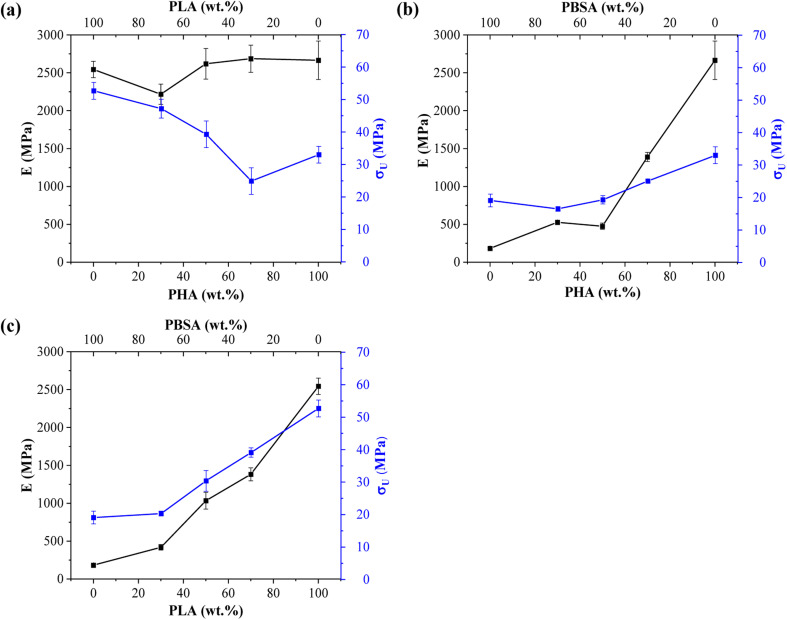
Changes in elastic modulus (*E*) and ultimate tensile strength (*σ*) for binary blends: (a) PHA/PLA, (b) PHA/PBSA, and (c) PLA/PBSA.

**Fig. 5:**
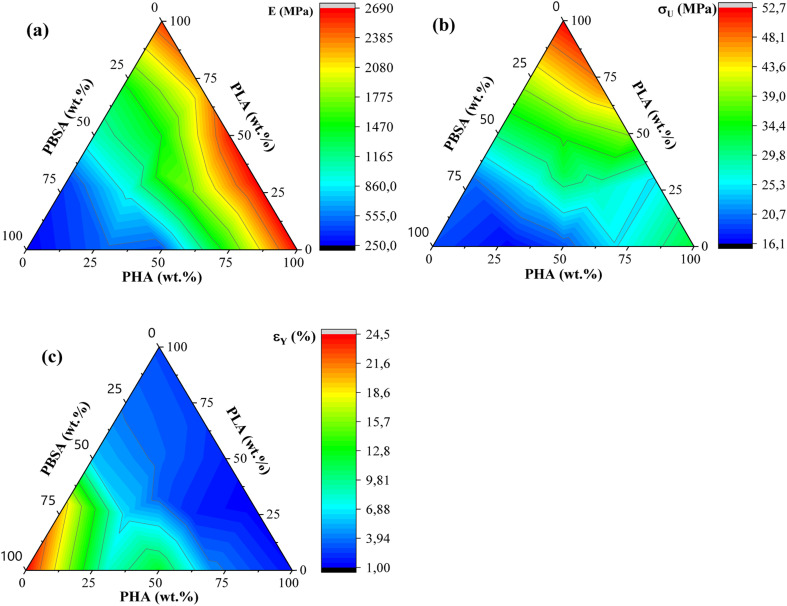
(a) Ternary diagrams for elastic modulus, (b) tensile strength (*σ*_U_), and (c) yield elongation (*ε*_Y_) for PHA/PLA/PBSA blends.

Tensile measurements were used to characterize blended polymer compatibility and understand the ability to tune the properties of bioplastics. Representative stress–strain curves are presented in Fig. S1.† At the same time, measured values such as elastic modulus (*E*), ultimate tensile strength (*σ*_U_), yield elongation (*ε*_Y_), and elongation at break (*ε*_B_) are visible in Table 2. Fig. 4 provides a visual comparison for changes in elastic modulus and ultimate tensile strength for binary blends and Fig. 5 shows ternary diagrams detailing the distribution of properties.

PLA showed the highest measured tensile strength of 52.7 MPa, about 1.6- and 2.8-fold higher than neat PHA and PBSA values, respectively. None of the blends showed higher values compared to neat polymers. This indicates that no inherent synergy was observed between the blended polymers. The highest values for blends responded to the highest PLA content, while the lowest ones responded to the highest PBSA content. Some of the blend ratios showed a notable drop in tensile strength, which was observed for A7L3, A7S3, and A2LS. This observation indicates that PHA is unsuitable as the primary matrix component and should be used in lower ratios. PLA and PBSA blends showed excellent ability to scale their tensile strength, with PLA even slightly enhancing the overall strength value for L3S7, where PBSA is the polymer matrix.

In the case of elastic modulus, PHA had the highest value of 2666 MPa, closely followed by PLA's 2545 MPa, but PBSA showed a 10-fold lower value of 250 MPa. The contribution from individual polymers was observable for blends. Unfortunately, PHA's low elongation and tensile strength values resulted in significant drawbacks for its binary blends with PBSA compared to PLA/PBSA blends. PBSA worked as intended, providing enhanced elongation and yielding blends with properties between neat polymer blends. For example, L5S5 showed a 4-fold improvement in tensile strength and a 1.6-fold increase in tensile strength compared to PBSA while also showing a 2-fold improvement in yield elongation compared to neat PLA. At the same time, A5S5 showed relatively low compatibility in this ratio, showing very similar values to those of A3S7. Compared to other binary blends, the PLA and PBSA blends are the most consistent of the three binary systems.

Most samples showed brittle failure, while PBSA and some of its blends had pronounced plastic deformation with elongation at break values up to 320%. For this reason, two elongation values are presented: yield elongation and elongation at break. PLA showed about 3-fold higher elongation at break value than PHA's 1.5%. Relatively low elongation measured for ternary blends in tensile tests did not show advantages to forming these blend ratios from selected polymers.

ALS2 stands out with properties similar to L5S5. While remaining ternary blends show higher elastic modulus than somewhat similar A7S3 and L7S3 but with lower elongation values. Looking at the tensile strength values, ternary blends show values somewhere between two similar polymer blends. For example, L5S5 has a 30.4 MPa tensile strength, and A5S5 has 19.3 MPa, while similar content ternary system ALS2 has a tensile strength of 24.6 MPa. Unlike binary blends, none of the ternary blends stand out, with a significant drop in properties at tested ratios.

Liu *et al.* reported that blending PBS/PLA and PBSA/PLA in the range from 80/20 and 20/80 blends.^24^ The authors noted that only 20 wt% of PBS or PBSA provides notable increase of elongation at break to more than 200%, changing the brittle behaviour to ductile. Zhang *et al.* reported that PLA/PHB blends with ratios of 25/75, 50/50, and 75/25 exhibited brittle fracture.^25^ Additionally, increasing the concentration of PLA improved the tensile strength and elongation at break. For the 75/25 and 50/50 blends, the tensile strength and elongation at break were lower than those of neat PLA. However, the 75/25 blend, where PLA is the continuous phase, exhibited better mechanical properties than pure PLA. Peshne *et al.* reported that blending PHB or PHBV with PBS decreases the blends' tensile strength and tensile modulus, while the elongation at break increases with the addition of PBS.^26^ Righetti *et al.* reported that blending PHB with PBS or PBSA changes the fracture behavior from brittle to ductile when the concentration of PHB is minimized.^27^ Specifically, PHB/PBS and PHB/PBSA blends with an 85/15 ratio did not exhibit necking formation. Conversely, necking was observed in blends with ratios of 70/30 and 50/50. Additionally, PBSA is characterized by higher chain mobility compared to PBS. Therefore, PBSA-rich blends are characterized by strain hardening, likely due to chain alignment.

The tensile properties of the blends can be correlated with the microstructure discussed in the previous section. Specifically, droplet morphology is well-documented for its ability to enhance the toughness and ductility of polymer blends.^19,28^ The soft and flexible regions formed by the ductile polymer (PBSA) serve to absorb mechanical energy, aiding in the dissipation of stress. Blend compositions that could exhibit this mechanism include A7S3, L7S3, A2LS, and AL2S. Among these, the L7S3 blend shows a clear droplet morphology, with a notable increase in elongation at break compared to neat PLA.

### 3.3. Impact strength

**Fig. 6:**
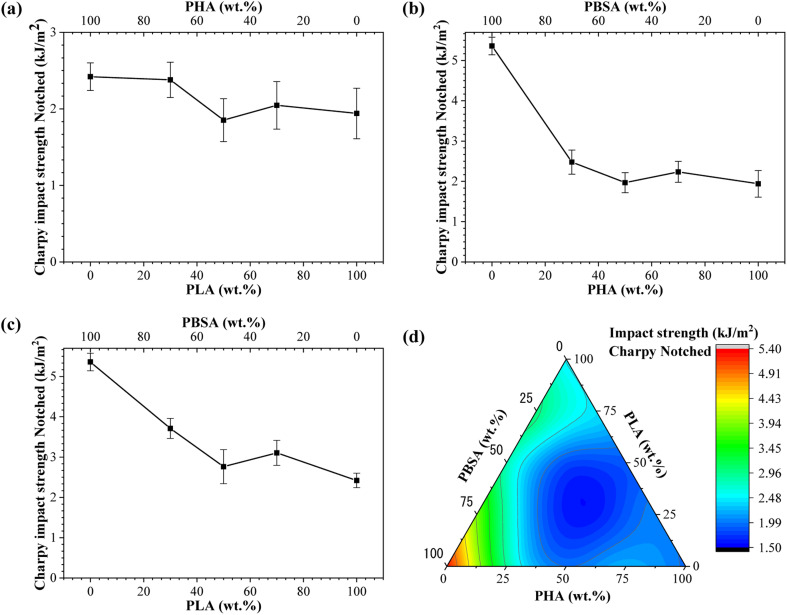
Charpy impact strength notched for (a) PLA/PHA, (b) PHA/PBSA, (c) PLA/PBSA binary blends. (d) Ternary diagram of impact strength distribution between PHA/PLA/PBSA blends.

Impact strength of neat polymers and blends processed by injection molding is shown in Fig. 6. These characteristics are related to its overall toughness and they are susceptible to the cohesion and miscibility of the blends. The impact strength data of binary blends show that PBSA is the most significant component. Neat PBSA is characterized by the highest impact strength value of 5.36 kJ m^−2^, which is about 3- and 2-fold higher than PHA and PLA, respectively. The impact strength of PHA/PBSA and PLA/PBSA blends increased with PBSA content, with notable exception for ratio 5/5. Similarly, PHA/PLA blend with a ratio of 5/5, demonstrated the lowest impact strength value of 1.85 kJ m^−2^. This could be attributed to the lack of continuous matrix from single polymer.

The impact strength of A3S7 blend was more than 1.3-fold higher in compression with pure PHA, while L3S7 demonstrated a 1.5-fold increase compared to neat PLA. In addition, PHA/PBSA impact strength values varied from 1.97 to 2.48 kJ m^−2^, while for PLA/PBSA, there were 2.76–3.71 kJ m^−2^. From examined blends, PLA/PBSA compositions show the best scaling of impact strength, while only L3S7 showed very pronounced plastic deformation in a tensile test. The observed increase in elongation at break for L7S3, characterized by droplet morphology, did not correspond to significant improvements in impact strength, which may be attributed to limited phase compatibility. From PHA/PLA blends notable was A3L7, which achieved 2.38 kJ m^−2^ and was comparable in performance to neat PLA. Zang *et al.* reported that the structure of PHA is highly affected by PLA, which leads to a slight increase in fracture toughness.^29^

In the case of ternary blends, the highest impact resistance was observed for ALS2, which corelates with the highest PBSA content. As observed from binary blends, the compositions with 50 wt% loading of components resulted in decreased impact strength, which matches to our formulation of ternary systems. Thus, unsurprisingly with lack of primary matrix, *i.e.*, single component above 50 wt%, ternary systems underperformed.

### 3.4. Viscoelastic characterization *via* dynamic mechanical analysis

**Fig. 7:**
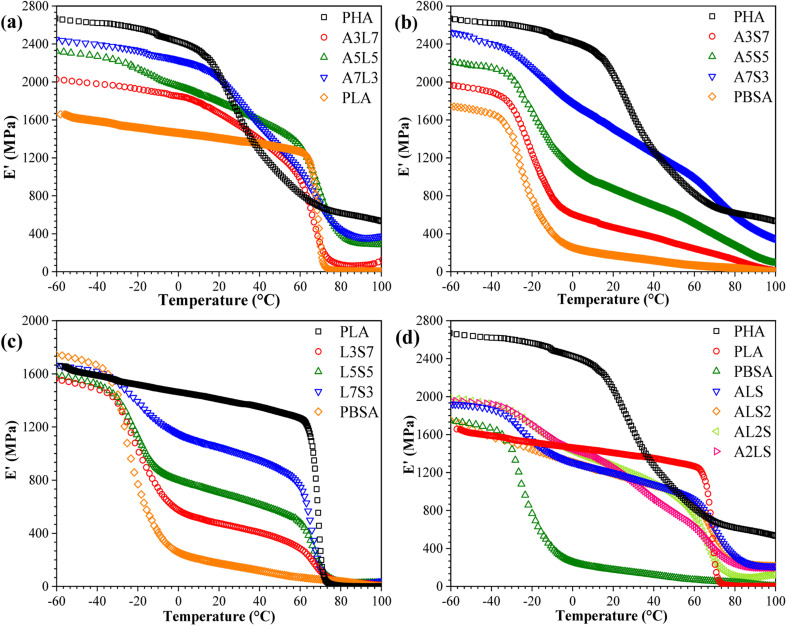
Storage modulus (*E*′) as a function of temperature for (a) PHA/PLA, (b) PHSA/PBSA, (c) PLA/PBSA, and (d) PHA/PLA/PBSA blends.

The storage modulus (*E*′) of neat PHA, PLA, PBSA, and their binary and ternary blends as a function of temperature is presented in Fig. 7. The storage modulus exhibits a trend analogous to the tensile properties, indicating a scaling of properties among the selected polymers. The samples demonstrate a typical decline in *E*′, which is attributed to increased macromolecular chain mobility and weaker intermolecular bonding.^30^ PHA displayed the highest storage modulus of 2600 MPa at −60 °C, whereas PLA and PBSA exhibited values of approximately 1600 MPa and 1700 MPa, respectively. PBSA has a relatively low glass transition temperature (*T*_g_), around −20 °C, whereas PLA and PHB remain glassy at room temperature. Consequently, the incorporation of PBSA into PHA/PBSA and PLA/PBSA blends results in a lower storage modulus above its *T*_g_. In PHA/PLA blends, a more pronounced difference is observed between the storage modulus when compared to the tensile elastic modulus. The glass transition of PLA occurs within a relatively low temperature range, whereas PHA exhibits a glass transition starting around 20 °C and extending to the measured 100 °C. This observation suggests that commercial PHA grades may contain a mixture of polymers with a broad molecular weight distribution. Additionally, this could be attributed to the thermal degradation of PHA during processing, which produces a polymer mixture. In the case of the ternary blends (Fig. 7(d)), remarkably similar performance was observed across all compositions. This similarity is attributed to the absence of a continuous matrix, resulting in a uniform impact of the glass transition temperatures of each component.

**Fig. 8:**
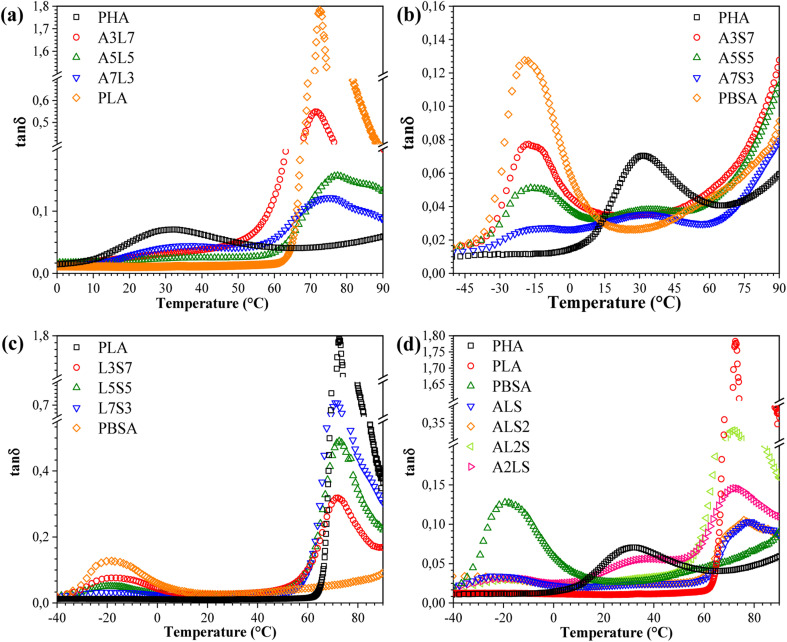
Loss factor (tan *δ*) as a function of temperature for (a) PHA/PLA, (b) PHA/PBSA, (c) PLA/PBSA, and (d) PHA/PLA/PBSA blends.

The tan *δ* peaks are indicative of transitions in molecular mobility, which correspond to the polymer materials' *T*_g_. Plotting the tan *δ* of a blend can elucidate the miscibility of the blend system. The behavior of tan *δ* as a function of temperature for binary and ternary blends is depicted in Fig. 8. A pronounced peak was observed around 72 °C for neat PLA, corresponding to its *T*_g_. The presence of two glass transitions in the tan *δ* curves signifies that the blends are not thermodynamically miscible. Notably, the *T*_g_ of PHA shifted to higher temperatures with increasing PLA content, while the *T*_g_ of PLA in the blends was lower than that of neat PLA and remained nearly unchanged with varying PLA contents. These variations in *T*_g_ with PLA content suggest an intramolecular compositional interaction between PLA and PHA in the amorphous region, which enhances the compatibility of the PHA/PLA blends.^31^ In other words, the blends composed of PLA and PHA exhibited partial miscibility. For PLA/PBSA blends (Fig. 7(c)), two distinct peaks at approximately −20 °C and 80 °C represented the glass transition temperatures of PBSA and PLA, respectively. Concurrently, the glass transition shifted towards lower temperatures, which could be related to the constrained movement of polymer chains.^30^ Additionally, the amplitude of PLA tan *δ* increased, while the peak height decreased with the addition of PBSA, suggesting that PBSA acts as a plasticizer, thereby reducing the stiffness of the blends.

In the case of the ternary blends, increasing the PHA and PBSA content shifted the tan *δ* peak toward lower temperatures, indicating reduced miscibility between the components. Adjusting the concentrations of PHA and PLA in the blends caused a more prominent shift in their respective *T*_g_'s towards each other, compared to the *T*_g_ of PBSA. This result demonstrates that PHA and PLA exhibit stronger interfacial interactions.^32^ Additionally, the reduced peak height can be attributed to the toughening effect caused by the presence of PBSA in the ternary blends. This finding demonstrates the potential for achieving a balance between toughness and stiffness by fine-tuning the relative amounts of each polymer in the blend.

### 3.5. Calorimetric properties

**Fig. 9:**
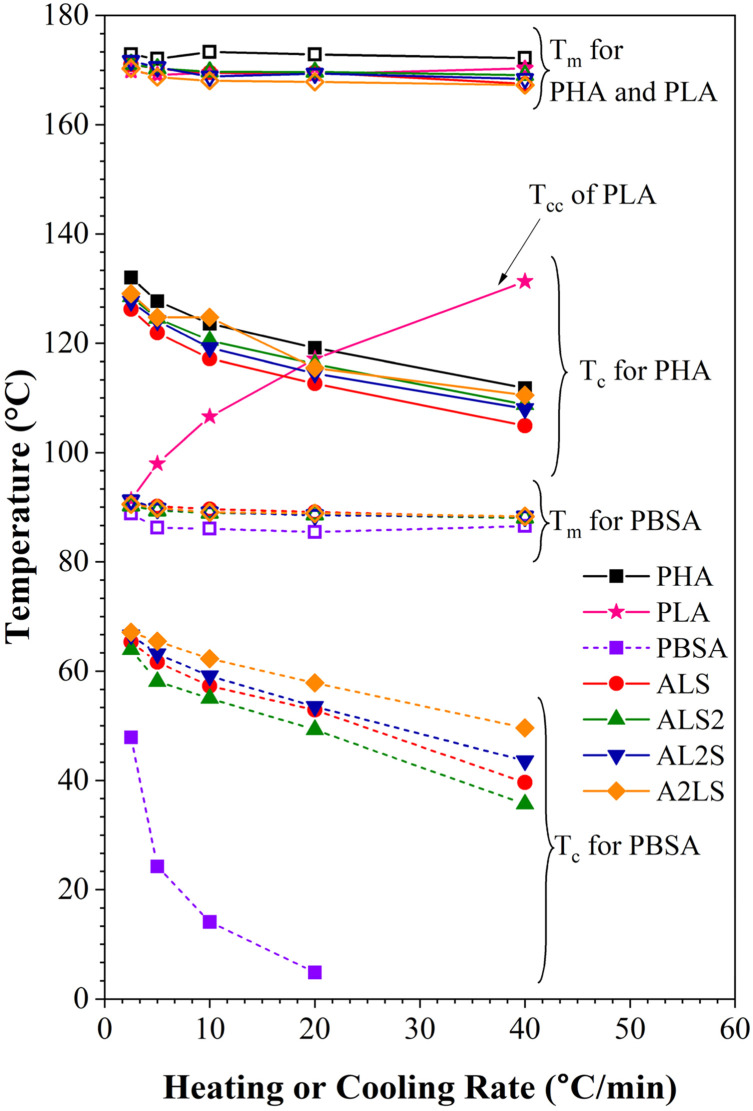
Melting and crystallization temperatures as a function of heating and cooling rates for neat polymers and ternary blends.

The thermal properties of the polymer blends can be significantly affected by the crystallization characteristics of individual polymers as well as the cooling or heating rate. Fig. S2–S8† shows DSC cooling and second heating scans for PHA, PLA, and PBSA and their ternary blends under five different cooling/heating rates. The obtained crystallization and melting values for neat polymers and ternary blends are listed in Tables S1–S6,† which include crystallization (*T*_c_), cold crystallization (*T*_cc_) and melting (*T*_m_) temperatures, and crystallinity (*χ*%). The crystallization temperatures and melting temperatures for pure PHA, PLA, and PBSA polymers and their ternary blends were plotted in Fig. 9 as a function of heating or cooling rate.

As seen in Fig. S2–S8† and 9, the crystallization peaks broaden and shift toward lower temperatures as the cooling rate increases. The polymers in the blends did not have time to orient their molecules for crystallization as the cooling rate increased. As a result, the nucleation shifted to a lower temperature. In addition, as the crystallization rate increases, polymers are characterized by a decrease in the size of crystals with a broad size distribution.^33,34^

It is worth noting that the *T*_c_ of PHA in the blends is lower than the neat PHA, while for PBSA, all ternary blends increase the crystallinity of PBSA (Tables S1–S5†). This reduction of PHA *T*_c_ can be attributed to the presence of melted PBSA and amorphous PLA domains, which can interfere with the crystal growth, restrict the phase, and induce nucleation suppression.^16,35^ Crystallization of PBSA turned out to be favored and can be linked to the existence of PHA spherulites, which can act as nucleation points for PBSA crystals.^27,36^

As for the melting peak, an increase in the heating rate shifts the peaks to lower temperatures. When PHA, PLA, and PBSA do not have time to crystallize at the high cooling rate and the size of the crystalline phase decreases, a drop in *T*_m_ with the rising heating rate is observed due to the formation of the imperfect crystalline phase.

When PLA is not crystallized during cooling, it's cold crystallization (*T*_cc_) is observed during the second heating. The curves shown in Fig. S3† (also, Fig. 9) indicate that increasing the heating or cooling rates, PLA *T*_cc_ shifted to higher temperatures, while the second exothermic peak became more intense.

The rise in *T*_cc_ can be explained by the fact that at higher heating rates, there is not enough time for it to crystallize at lower temperatures. As a result, this fraction undergoes cold crystallization at higher temperatures just before the main melting endotherm. Conversely, there is ample time for nucleation and crystalline growth at lower heating rates, facilitating the formation of larger and more perfect crystals. Table S6(a)† shows that one exothermal peak for neat PLA at the cooling rate of 2.5 °C min^−1^ is observable.

**Fig. 10:**
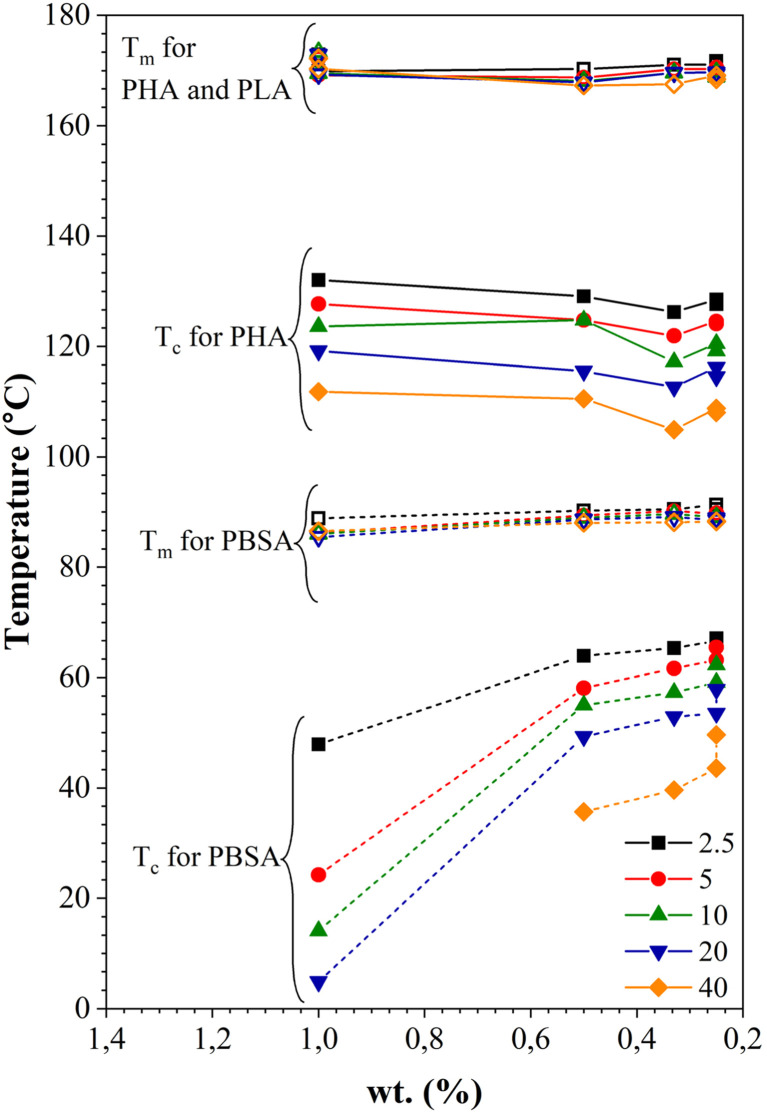
Dependence of melting and crystallization temperatures on the weight fraction (wt%) of PHA, PLA, and PBSA at various heating and cooling rates.

Fig. 10 shows the dependence of *T*_c_ and *T*_m_ on the PHA, PLA, and PBSA concentration at various heating and cooling rates. The increase of *T*_c_ is more pronounced for PBSA. Decreased concentration of PBSA in the blends results in its melting temperature rising at all cooling rate ranges. The decrease of the crystallization temperature can be caused by the dilution effect, slowing down both growth of crystals and homogeneous nucleation. The *T*_c_ of PHA remains relatively constant. The drop of *T*_c_ was visible for all crystallization rates when the PHA weight fraction was 33 wt%. This may indicate that PBSA and/or PLA hinder the crystallization process of PHA. The melting temperatures for both PBSA and PHA remain relatively constant or shift to a higher *T*_m_.

## 4. Conclusions

This study investigated the potential of polymer blends to tailor the mechanical properties of bioplastics derived from PLA, PHA, and PBSA. PLA/PBSA blends exhibited superior mechanical properties and consistency compared to PHA/PBSA blends, demonstrating that PLA and PBSA are more compatible for creating mechanically robust bioplastics.

In the binary blends, droplet inclusions were the predominant morphology at a 3 : 7 ratio, while a co-continuous morphology was dominant at a 5 : 5 ratio. Distinct morphological variations were observed between compositions, which can be correlated with differences in mechanical performance. The ternary blends exhibited rougher surfaces with more irregular inclusions and complex, mixed morphologies. The density of the blends remained relatively consistent with that of the neat polymers, with only minor deviations, suggesting minimal structural defects or changes in crystallinity.

PLA demonstrated the highest tensile strength among the neat polymers, and its blends with PBSA showed enhanced tensile strength and elongation compared to other binary blends. However, no inherent synergy was observed between the blended polymers. PBSA improved the impact strength of the blends. PLA/PBSA blends showed the best scaling of impact resistance, while PHA/PBSA blends showed relatively poor performance.

The storage modulus of the blends followed a trend consistent with the tensile properties, with PBSA contributing to a lower modulus due to its viscoelastic nature. The tan *δ* curves indicated partial miscibility for PLA/PHA blends and poor miscibility for other binary and ternary blends. The glass transition temperatures in PBSA binary blends remained largely independent of the blend composition. The crystallization temperature showed a significant increase for PBSA in the blends, while the inverse effect on PHA was less pronounced. Both the scan rate and polymer concentration visibly influenced the crystallization behavior. Blends in which none of the components exceeded 50 wt% generally exhibited the lowest properties. Future research should focus on optimizing blend compositions, *i.e.*, increasing the major component content, and exploring additional compatibilization methods to further improve the properties and applicability of bioplastic blends.

## Data availability

The data supporting this article have been included as part of the ESI.†

## Author contributions

Alisa Sabalina: data curation; formal analysis; validation; visualization; writing – original draft. Oskars Platnieks: supervision, writing – original draft; writing – review and editing. Gerda Gaidukova: resources, validation. Arturs Aunins: data curation; formal analysis; investigation; validation; visualization. Toms Valdemars Eiduks: investigation. Sergejs Gaidukovs: conceptualization, methodology; resources; supervision; writing – review and editing.

## Conflicts of interest

The authors declare that they have no known competing financial interests or personal relationships that could have appeared to influence the work reported in this paper.

## Supplementary Material

RA-015-D4RA05684A-s001
