# Supplementary material for: Thermomechanical and mechanical analysis of polylactic acid/polyhydroxyalkanoate/poly(butylene succinate-co-adipate) binary and ternary blends
Source: RSC Adv. 2025 Jan 6;15(1):501–12. doi: 10.1039/d4ra05684a (PMC11701805; doi:10.1039/d4ra05684a)
Supplement: RA-015-D4RA05684A-s001 [file RA-015-D4RA05684A-s001.pdf]

Supplementary Information (SI) for RSC Advances.
This journal is © The Royal Society of Chemistry 2024

Supplementary Information

Thermomechanical and Mechanical analysis of Polylactic acid / Polyhydroxyalkanoate / Poly(butylene succinate-*co*-adipate) binary and ternary blends

Alisa Sabalina [a], Oskars Platnieks [*a], Gerda Gaidukova [a], Arturs Aunins [a], Toms Valdemars Eiduks [b], Sergejs Gaidukovs [*a]

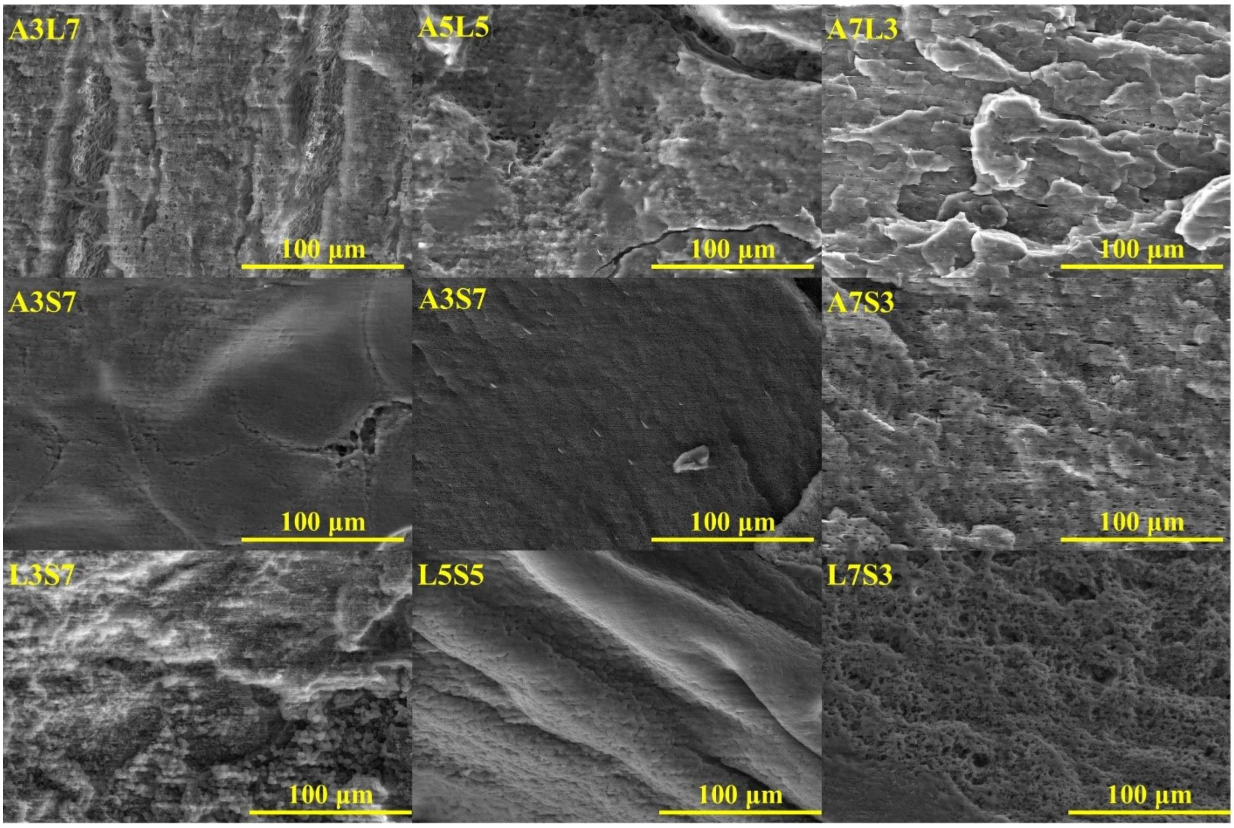

Figure S1. SEM micrographs of binary blends.

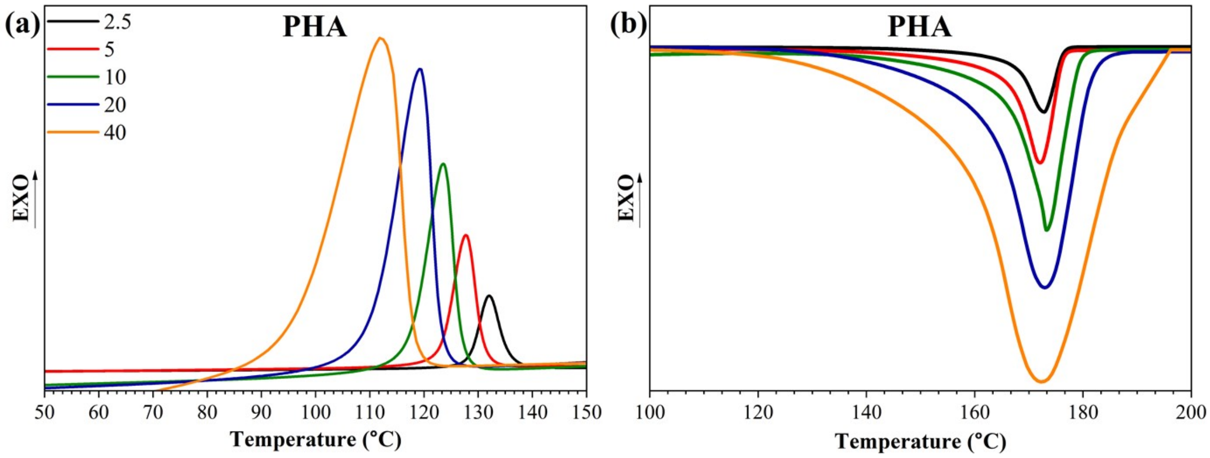

Figure S2. DSC (a) cooling and (b) second heating scans of neat PHA.

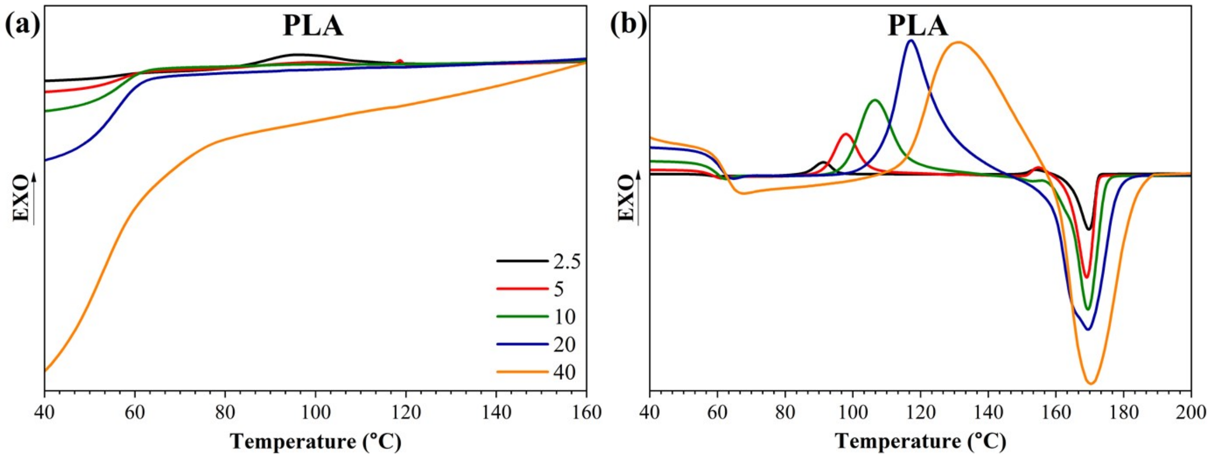

Figure S3. DSC (a) cooling and (b) second heating scans of neat PLA.

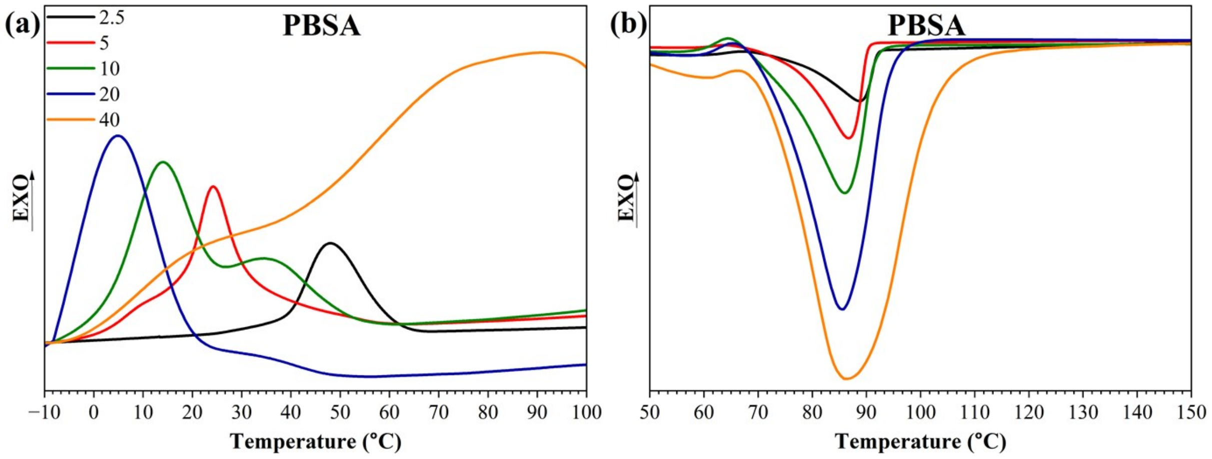

Figure S4. DSC (a) cooling and (b) second heating scans of neat PBSA.

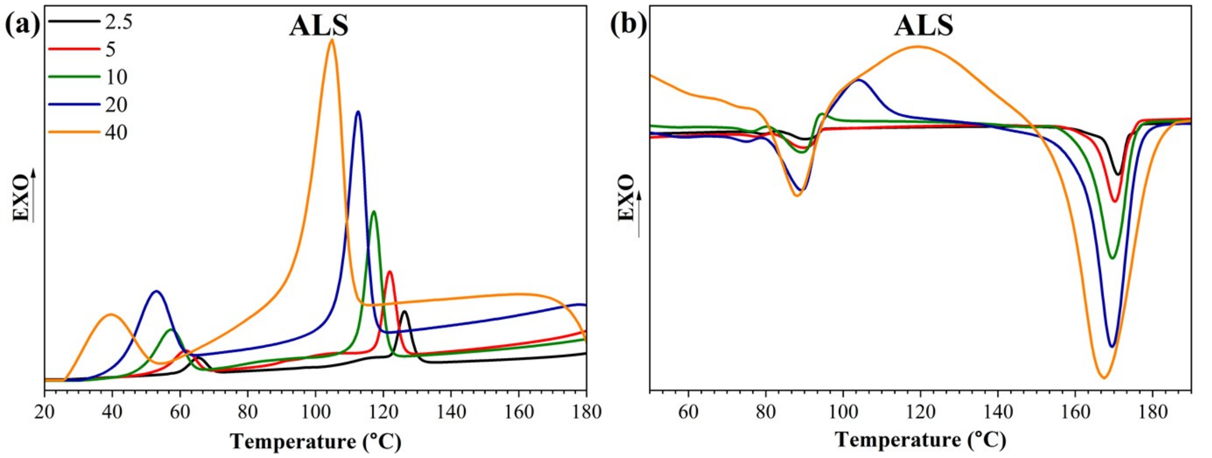

Figure S5. DSC (a) cooling and (b) second heating scans of ALS blend.

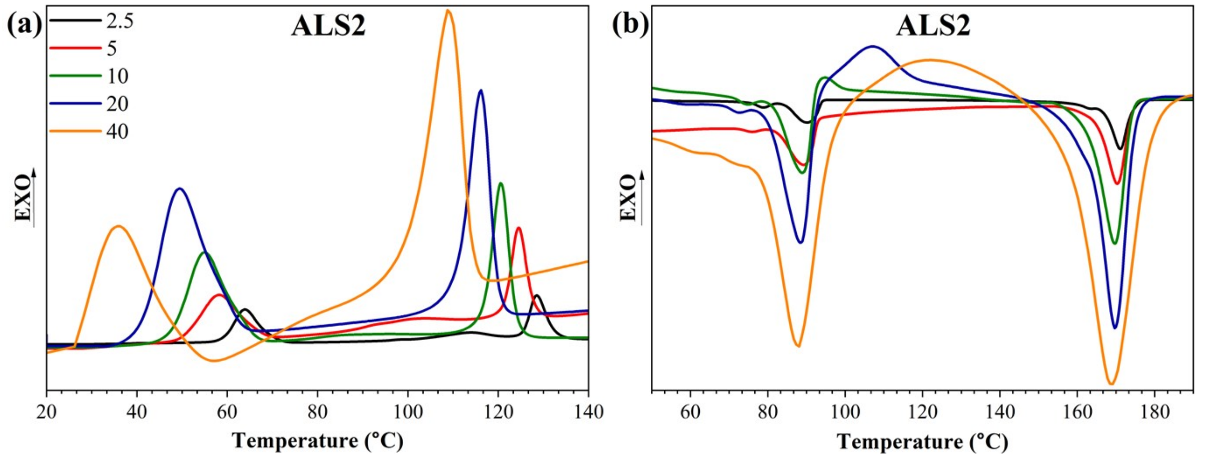

Figure S6. DSC (a) cooling and (b) second heating scans of ALS2 blend.

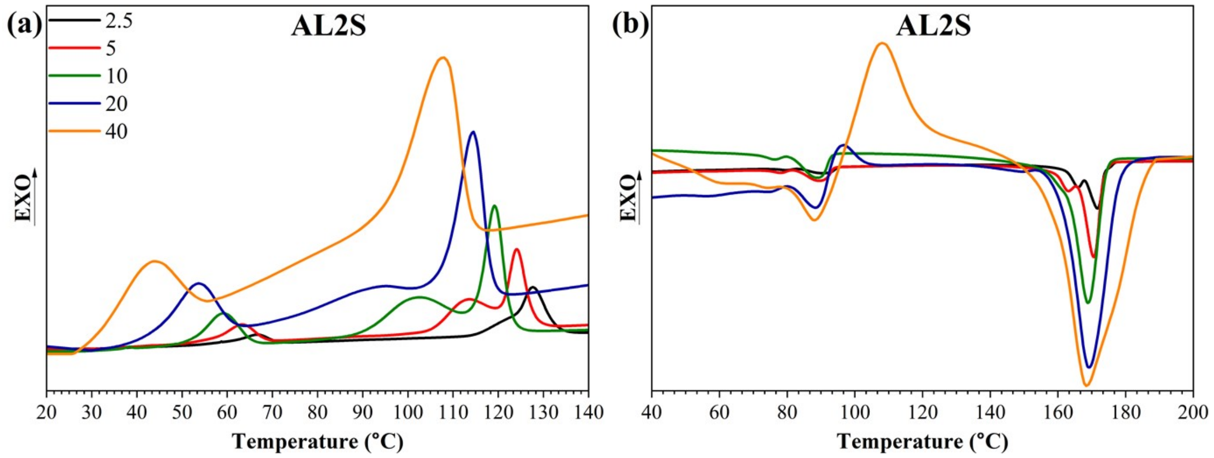

Figure S7. DSC (a) cooling and (b) second heating scans of AL2S blend.

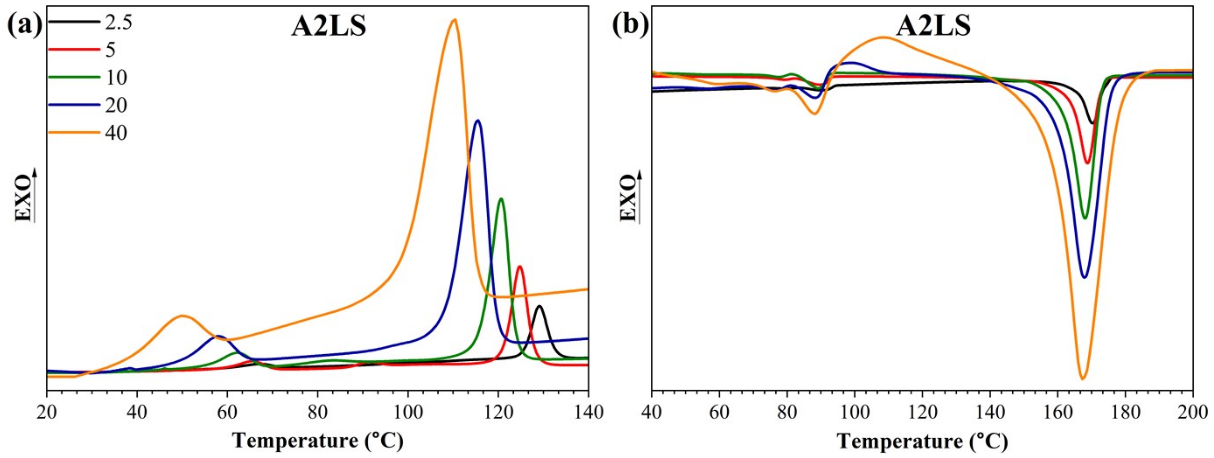

Figure S8. DSC (a) cooling and (b) second heating scans of A2LS blend.

Table S1. Thermal properties of ternary PHA, PLA, and PBSA blends with a heating rate of 2.5 ºC/min.

Sample		T_c, ˚C	ΔH_c, J/g	χ,%	T_m, ˚C	ΔH_m, J/g
PHA		132.0	97.2	67	172.9	103.8
PLA		91.3	12.3	13	169.9	47.7
PBSA		47.9	47.0	43	88.8	45.8
ALS	PHA	126.2	34.0	71	171.1	38.6
	PLA	–	–	–	171.1*	–
	PBSA	65.3	11.6	32	90.5	8.4
ALS2	PHA	128.5	17.5	48	171.1	32.3
	PLA	–	–	–	171.1*	–
	PBSA	63.9	21.5	39	90.2	18.6
AL2S	PHA	127.7	44.0	121	171.7	47.9
	PLA	–	–	–	171.7*	–
	PBSA	66.6	8.1	29	91.3	5.6
A2LS	PHA	129.1	44.4	61	170.3	57.3
	PLA	–	–	–	170.3*	–
	PBSA	67.1	5.4	20	90.5	4.2

*PHA and PLA melting peaks overlay.

Table S2. Thermal properties of ternary PHA, PLA, and PBSA blends with a heating rate of 5 ºC/min.

Sample		T_c, ˚C	ΔH_c, J/g	χ,%	T_m, ˚C	ΔH_m, J/g
PHA		127.7	91.4	63	172.1	100.6
PLA		98.0	25.1	27	169.1	44.6
PBSA		24.3	37.2	34	86.3	41.3
ALS	PHA	121.9	22.8	47	170.3	37.4
	PLA	–	–	–	170.3*	–
	PBSA	61.7	10.6	29	90.2	8.3
ALS2	PHA	124.6	18.5	51	170.3	31.3
	PLA	–	–	–	170.3*	–
	PBSA	58.1	21.4	38.9	89.4	19.8
AL2S	PHA	124.1	41.1	113	170.6	45.9
	PLA	–	–	–	170.6*	–
	PBSA	63.2	8.2	30	89.7	5.8
A2LS	PHA	124.8	41.6	57	168.8	60.7
	PLA	–	–	–	168.8*	–

	PBSA	65.5	5.4	19.8	89.8	3.9

*PHA and PLA melting peaks overlay.

Table S3. Thermal properties of ternary PHA, PLA, and PBSA blends with a heating rate of 10 ºC/min.

Sample		T_c, ˚C	ΔH_c, J/g	χ,%	T_m, ˚C	ΔH_m, J/g
PHA		123.6	86.7	59	173.3	93.7
PLA		106.6	32.6	35	169.5	36.7
PBSA		14.1	28.1	26	86.1	41.9
ALS	PHA	117.2	24.7	51	169.6	39.7
	PLA	–	–	–	169.6*	–
	PBSA	57.23	11.9	33	89.6	8.1
ALS2	PHA	120.5	18.2	50	169.7	30.5
	PLA	–	–	–	169.7*	–
	PBSA	55.0	21.9	40	88.9	20.2
AL2S	PHA	119.2	37.5	103	168.8	44.4
	PLA	–	–	–	168.8*	–
	PBSA	59.1	7.5	27	89.1	5.7
A2LS	PHA	120.6	40.9	56	168.1	57.5
	PLA	–	–	–	168.1*	–
	PBSA	62.3	4.7	17	89.1	4.0

*PHA and PLA melting peaks overlay.

Table S4. Thermal properties of ternary PHA, PLA, and PBSA blends with a heating rate of 20 ºC/min.

Sample		T_c, ˚C	ΔH_c, J/g	χ,%	T_m, ˚C	ΔH_m, J/g
PHA		119.2	83.3	57	172.9	91.9
PLA		117.1	40.4	44	169.3	41.1
PBSA		4.9	16.6	15	85.5	42.6
ALS	PHA	112.6	23.7	49	169.6	40.1
	PLA	–	–	–	169.6*	–
	PBSA	52.9	11.1	31	89.1	6.2
ALS2	PHA	116.2	16.6	46	169.7	29.0
	PLA	–	–	–	169.7*	–
	PBSA	49.3	22.0	40	88.6	15.1

AL2S	PHA	114.5	15.4	42	169.4	38.3
	PLA	–	–	–	169.4*	–
	PBSA	53.5	7.4	27	88.5	2.3
A2LS	PHA	115.5	39.3	54	167.9	56.9
	PLA	–	–	–	167.9*	–
	PBSA	57.9	5.9	21	88.9	2.9

*PHA and PLA melting peaks overlay.

Table S5. Thermal properties of ternary PHA, PLA, and PBSA blends with a heating rate of 40 ºC/min.

Sample		T_c, ˚C	ΔH_c, J/g	χ,%	T_m, ˚C	ΔH_m, J/g
PHA		111.8	80.1	55	172.2	57.7
PLA		131.3	32.1	35	170.3	28.5
PBSA		–	–	–	86.6	36.4
ALS	PHA	104.9	21.7	45	167.5	34.3
	PLA	–	–	–	167.5*	–
	PBSA	39.6	6.3	17	88.1	6.8
ALS2	PHA	108.8	16.0	44	169.1	32.1
	PLA	–	–	–	169.1*	–
	PBSA	35.7	11.2	20	88.1	16.1
AL2S	PHA	108.0	14.2	39	168.4	33.0
	PLA	–	–	–	168.4*	–
	PBSA	43.6	5.8	21	88.3	2.8
A2LS	PHA	110.5	35.2	48	167.3	51.5
	PLA	–	–	–	167.3*	–
	PBSA	49.6	5.0	18	88.3	1.6

*PHA and PLA melting peaks overlay.

Table S6. Thermal properties of neat PLA at different heating or cooling rates.

Heating or cooling rate, ˚C/min	T_{cc}, ˚C	ΔH_{cc}, J/g	χ_{cc},%	T_m, ˚C	ΔH_m, J/g
2.5	91.3	12.3	13	169.9	47.7
5	97.9	25.13	27	169.1	44.6
10	106.5	32.6	35	169.5	36.7
20	117.1	40.4	44	169.3	41.1
40	131.3	32.1	34	170.3	28.5
